# Evaluation of the safety and efficacy of extracorporeal carbon dioxide removal in the critically ill using the PrismaLung+ device

**DOI:** 10.1186/s40001-023-01269-2

**Published:** 2023-08-18

**Authors:** Ravindranath Tiruvoipati, Jarryd Ludski, Sachin Gupta, Ashwin Subramaniam, Mallikarjuna Ponnapa Reddy, Eldho Paul, Kavi Haji

**Affiliations:** 1 Department of Intensive Care Medicine, Frankston Hospital, Peninsula Health, Frankston, VIC 3199 Australia; 2 Division of Medicine, Peninsula Clinical School, Monash University, Frankston, VIC Australia; 3 ANZIC-RC, School of Public Health and Preventive Medicine, Monash University, 553 St Kilda Road, Melbourne, VIC 3004 Australia; 4 Department of Intensive Care Medicine, Dandenong Hospital, Dandenong, Australia; 5 Department of Intensive Care, Calvary Hospital, Canberra, ACT Australia; 6 Alfred Hospital, Melbourne, VIC Australia; 7 Department of Surgery, University of Melbourne, Melbourne, VIC Australia

**Keywords:** Hypercapnia, Respiratory failure, Respiratory acidosis, Extracorporeal therapies, PrismaLung+

## Abstract

**Background:**

Several extracorporeal carbon dioxide removal (ECCO_2_R) devices are currently in use with variable efficacy and safety profiles. PrismaLung+ is an ECCO_2_R device that was recently introduced into clinical practice. It is a minimally invasive, low flow device that provides partial respiratory support with or without renal replacement therapy. Our aim was to describe the clinical characteristics, efficacy, and safety of PrismaLung+ in patients with acute hypercapnic respiratory failure.

**Methods:**

All adult patients who required ECCO_2_R with PrismaLung+ for hypercapnic respiratory failure in our intensive care unit (ICU) during a 6-month period between March and September 2022 were included.

**Results:**

Ten patients were included. The median age was 55.5 (IQR 41–68) years, with 8 (80%) male patients. Six patients had acute respiratory distress syndrome (ARDS), and two patients each had exacerbations of asthma and chronic obstructive pulmonary disease (COPD). All patients were receiving invasive mechanical ventilation at the time of initiation of ECCO_2_R. The median duration of ECCO_2_R was 71 h (IQR 57–219). A significant improvement in pH and PaCO_2_ was noted within 30 min of initiation of ECCO_2_R. Nine patients (90%) survived to weaning of ECCO_2_R, eight (80%) survived to ICU discharge and seven (70%) survived to hospital discharge. The median duration of ICU and hospital stays were 14.5 (IQR 8–30) and 17 (IQR 11–38) days, respectively. There were no patient-related complications with the use of ECCO_2_R. A total of 18 circuits were used in ten patients (median 2 per patient; IQR 1–2). Circuit thrombosis was noted in five circuits (28%) prior to reaching the expected circuit life with no adverse clinical consequences.

**Conclusion(s):**

PrismaLung+ rapidly improved PaCO_2_ and pH with a good clinical safety profile. Circuit thrombosis was the only complication. This data provides insight into the safety and efficacy of PrismaLung+ that could be useful for centres aspiring to introduce ECCO_2_R into their clinical practice.

## Background and rationale

Acute respiratory failure is one of the most common indications for admission of patients to the intensive care unit (ICU). Many of these patients require the assistance of invasive mechanical ventilation (IMV) in the management of respiratory failure. A strategy of preventing ventilator induced lung injury (VILI) by reducing inspiratory pressures and the driving pressure on IMV has been shown to reduce mortality [1, 2]. The current standard of care in treating patients with acute hypoxic respiratory failure is to use low tidal volume (< 6 ml/kg predicted body weight) ventilation [3]. One of the effects of such a ventilation strategy is the development of hypercapnia and related respiratory acidosis. Several recent studies have highlighted the adverse effects of hypercapnia when associated with lung protective ventilation [4–8]. Based on the evidence from these studies, hypercapnia should be avoided or actively treated when associated with lung protective ventilation.

There are several minimally invasive extracorporeal carbon dioxide removal (ECCO_2_R) devices that are currently available for the management of patients with severe hypercapnic respiratory failure [9–13]. Most of these devices provide partial respiratory support as compared to extracorporeal membrane oxygenation (ECMO), where total respiratory support can be provided. These devices are mainly used to remove CO_2_ from the blood to provide lung protective ventilation [14, 15]. Most of these less invasive devices are efficient in clearing CO_2_ but do not provide significant oxygenation [12, 13, 16]. The cannulas used to access blood vessels are smaller (13–16 F) in low-flow ECCO_2_R devices but can be cannulas similar to ECMO in high-flow devices. The anticoagulation targets are similar to other extracorporeal devices, such as renal replacement therapy circuits and ECMO devices. A recent study, however, observed a higher incidence of bleeding and haemolysis with low-flow ECCO_2_R devices [17].

The use of minimally invasive ECCO_2_R devices was reported in several studies with satisfactory clearance of carbon dioxide [9–12, 18]. However, some studies reported a higher incidence of complications such as haemolysis, bleeding, and inadequacy of obtaining satisfactory carbon dioxide clearance with the use of low-flow devices ECCO_2_R devices [17, 19–21].

One of the newer devices is called PrismaLung+ [22], which was recently introduced to clinical practice. PrismaLung+ is a low flow venovenous ECCO_2_R device that can provide CO_2_ removal with or without simultaneously providing renal replacement therapy.

## Aims and objectives

This study aimed to evaluate the safety and efficacy of ECCO_2_R with PrismaLung+ in mechanically ventilated critically ill patients.

## Methods

### Ethics approval

The human research ethics committees of Peninsula Health reviewed the study proposal and waived the requirement for a full ethics committee application (QA/89504/PH-2022-330245). This was because the study was seen as a retrospective audit of data routinely collected for patient care and not experimental research. Consent from individual patients was not required, since the research was limited to the use of information previously collected during normal care and the patients were not identifiable.

All mechanically ventilated patients with acute or acute-on-chronic hypercapnic respiratory failure, managed with ECCO_2_R over a period of 6 months (March 2022 to September 2022) in our hospital were included.

### *ECCO*_*2*_*R with PrimaLung* + 

**Fig. 1:**
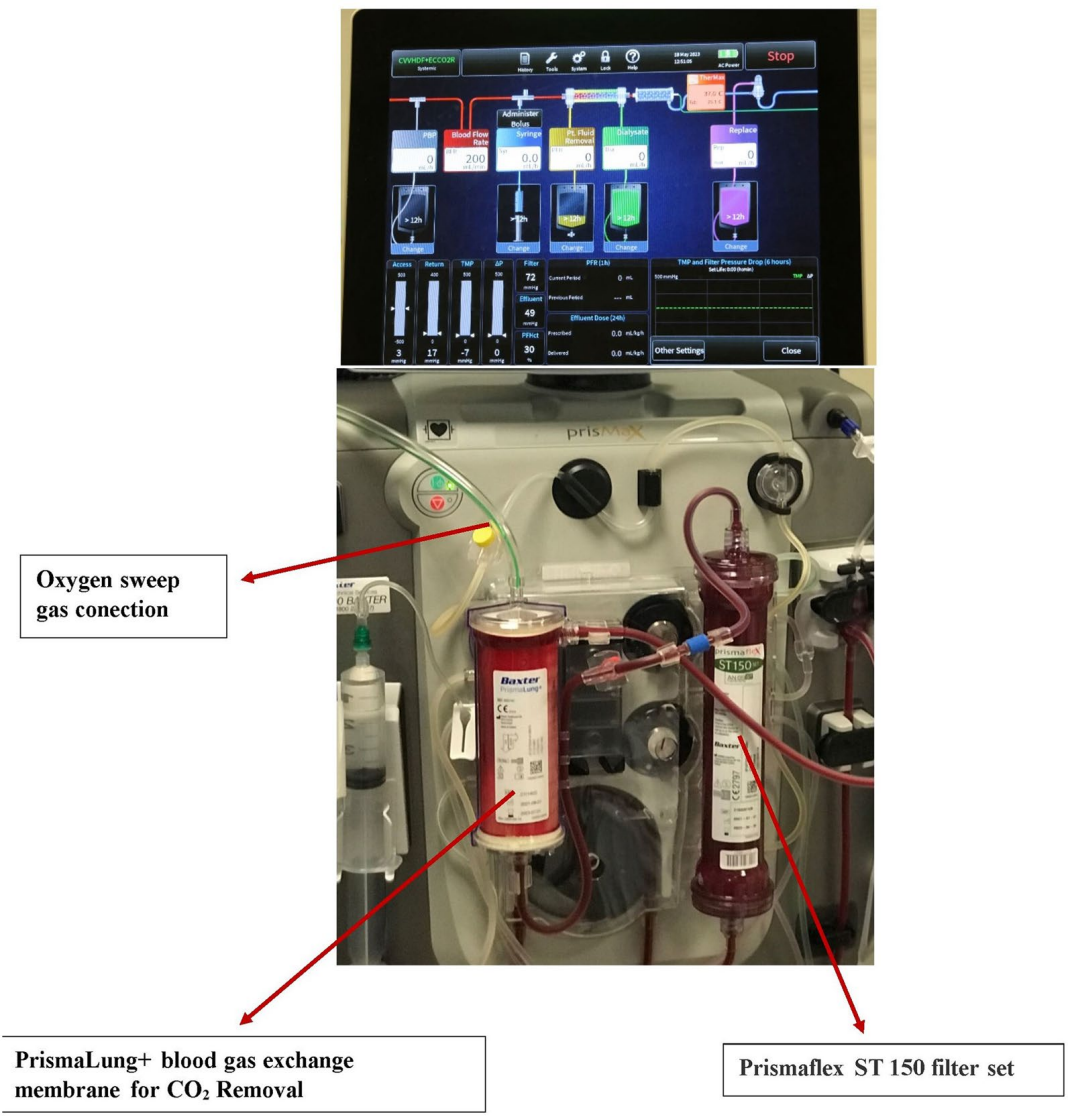
PrismaLung+

PrismaLung+ (Fig. 1) is a novel low flow venovenous device that integrates renal and respiratory extracorporeal supports. A detailed description of this device is provided elsewhere [23, 24]. It incorporates a gas exchange membrane made of polymethylpentene hollow-fiber mats, into Prismax (Baxter Healthcare Pty Ltd.) renal replacement system with or without the use of a haemofilter within the circuit [24]. PrismaLung+ has a larger gas exchange membrane surface area (0.8 m^2^) as compared to the earlier version of PrimaLung+ with a lower surface area (0.35 m^2^) making this a more effective device [24]. The total priming volume of the circuit is 273 mL.

### *Patient management on* ECCO_2_R

Access to blood flow was obtained by a 13F double-lumen catheter either via a femoral or jugular vein. Catheter insertion was performed using real-time ultrasound guidance. Heparin was used for anticoagulation, aiming for an Activated Partial Thromboplastin Time (APTT) of 50–70 s. Blood flow was established at a rate of 200 mL/min to 250 mL/min. Oxygen was used as sweep gas. The sweep gas flow was gradually increased to 10 L/min to provide effective ECCO_2_R. After lung recovery, ECCO_2_R weaning was initiated by down-titrating sweep gas flow and thereby reducing the amount of CO_2_ removal to zero. After confirming adequate respiratory function, ECCO_2_R was disconnected from the patient at the discretion of the treating intensivist.

Low tidal volume (≤ 6 mL/kg ideal body weight), and low-pressure ventilation were targeted for all patients included in this study, with no pre-specified protocol on the mode of IMV. Asthmatic patients were mechanically ventilated with a low tidal volume (5–6 mL/kg), a low respiratory rate (10–12 breaths/min), and a short inspiratory time associated with prolonged expiratory time to avoid dynamic hyperinflation.

## Indications for the use of ECCO_2_R

Patients were managed with ECCO_2_R at the discretion of the treating intensivist if the patient was receiving IMV but could not be ventilated with lung protective ventilation (tidal volumes ≤ 6 mL/kg of ideal body weight) due to hypercapnic respiratory failure (respiratory acidosis (pH < 7.25 and pCO_2_ > 55 mmHg).

## Contraindications to ECCO_2_R

- Contraindication for limited anticoagulation (Heparinisation to achieve an APTT of 50–70 s or an activated clotting time (ACT) of 150–180 s).
- Platelet count of less than 75,000/mm^3^.
- Patients who had established treatment limitations (i.e., not for cardiopulmonary resuscitation, or admitted to ICU for palliative care or organ donation purposes, not for intubation, mechanical ventilation, and not for continuous renal replacement therapy in ICU).

## Primary outcome measure

CO_2_ clearance and improvement in pH with the use of ECCO_2_R.

## Secondary outcome measures

- Complications associated with ECCO_2_R
- Survival to weaning from ECCO_2_R, ICU and hospital discharge.

Complications: Classified as patient-related or device-related.

Patient-related:

- Bleeding: Clinically significant bleeding that required blood transfusions, the need to stop anticoagulation, the need for surgery or any other interventions to stop bleeding during the ECCO_2_R
- Bleeding from the catheter site
- Intracranial bleeding
- Disseminated intravascular coagulation or thromboembolism
- Pneumothorax
- Cardiac arrhythmias
- Hypothermia
- Haemodynamic instability: Tachycardia or hypotension (< 90 mmHg of systolic blood pressure) at the commencement of ECCO_2_R that may be attributed to ECCO_2_R initiation.
- Catheter infection: Infection at catheter site that resulted in bacteraemia.
- Clinically significant haemolysis: Jaundice or anaemia that is not due to another recognisable cause.
- Severe thrombocytopenia (< 50,000/mm^3^).

Device-related:

- Circuit thrombosis*:* Clotting of membrane lung or the circuit that needed circuit replacement prior to reaching the expected circuit life (within 72 h of starting the circuit).
- Pump malfunction, inability to start ECCO_2_R or air in circuit.

### Statistical analysis

Categorical variables are presented as counts and percentages and continuous variables as medians and interquartile ranges (IQR). Changes in pH, PaCO_2_, PaO_2_, peak inspiratory pressure and minute ventilation from baseline values prior to initiation of PrismaLung+ and at successive time points were assessed and summarised using means and standard errors. To account for repeat measures, data were analysed using the PROC MIXED procedure in SAS with each patient treated as a random effect. Time was treated as a categorical variable to facilitate specific comparisons. A two-sided *P* < 0.05 indicated statistical significance. All analyses were performed using SAS software version 9.4 (SAS Institute, Cary, NC, USA) and SPSS version 22 (IBM SPSS, Armonk, NY).

## Results

**Fig. 2:**
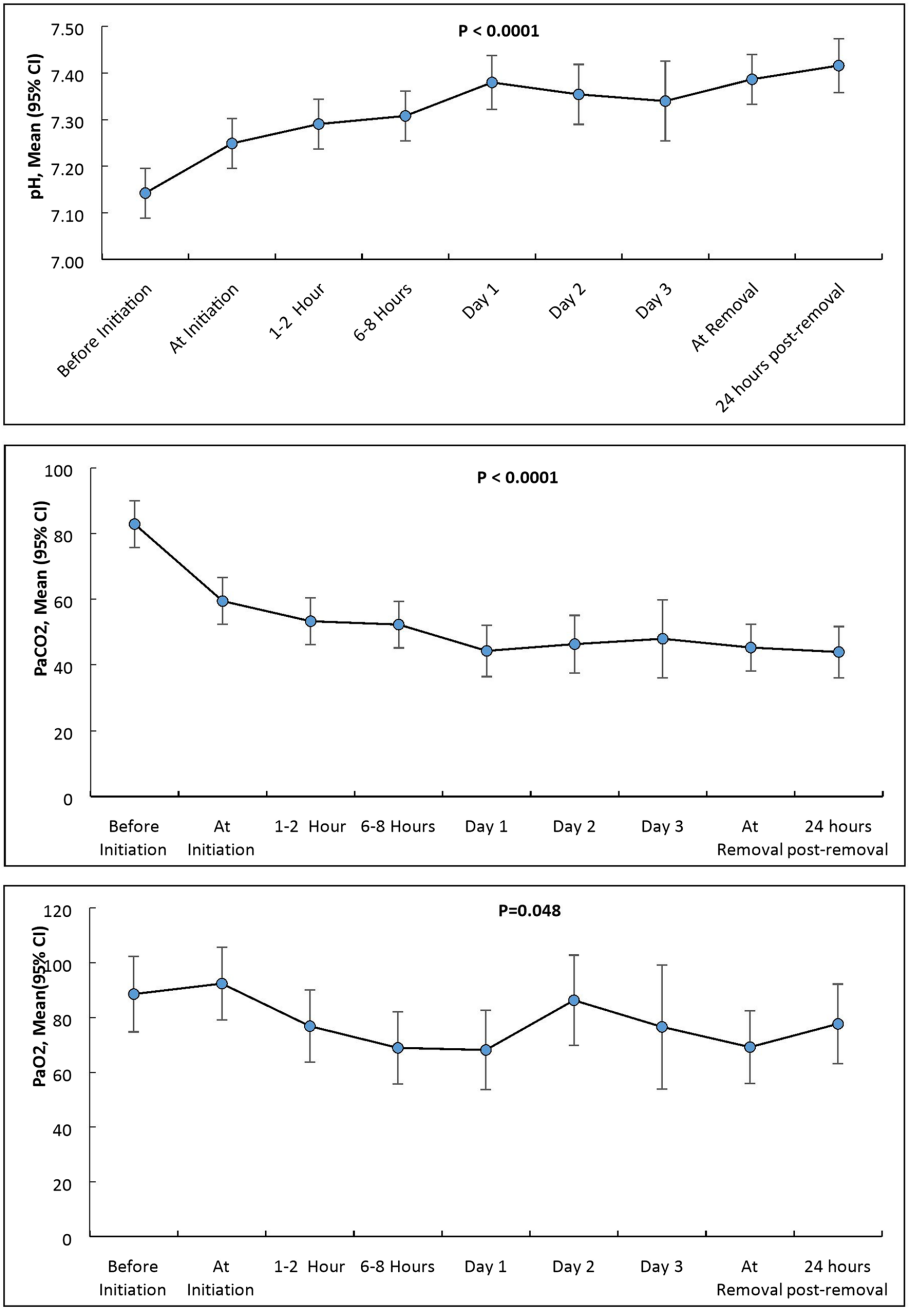
Changes in pH, PCO_2_ and PO_2_ before initiation and at successive time points. Error bars represent 95% confidence intervals

**Fig. 3:**
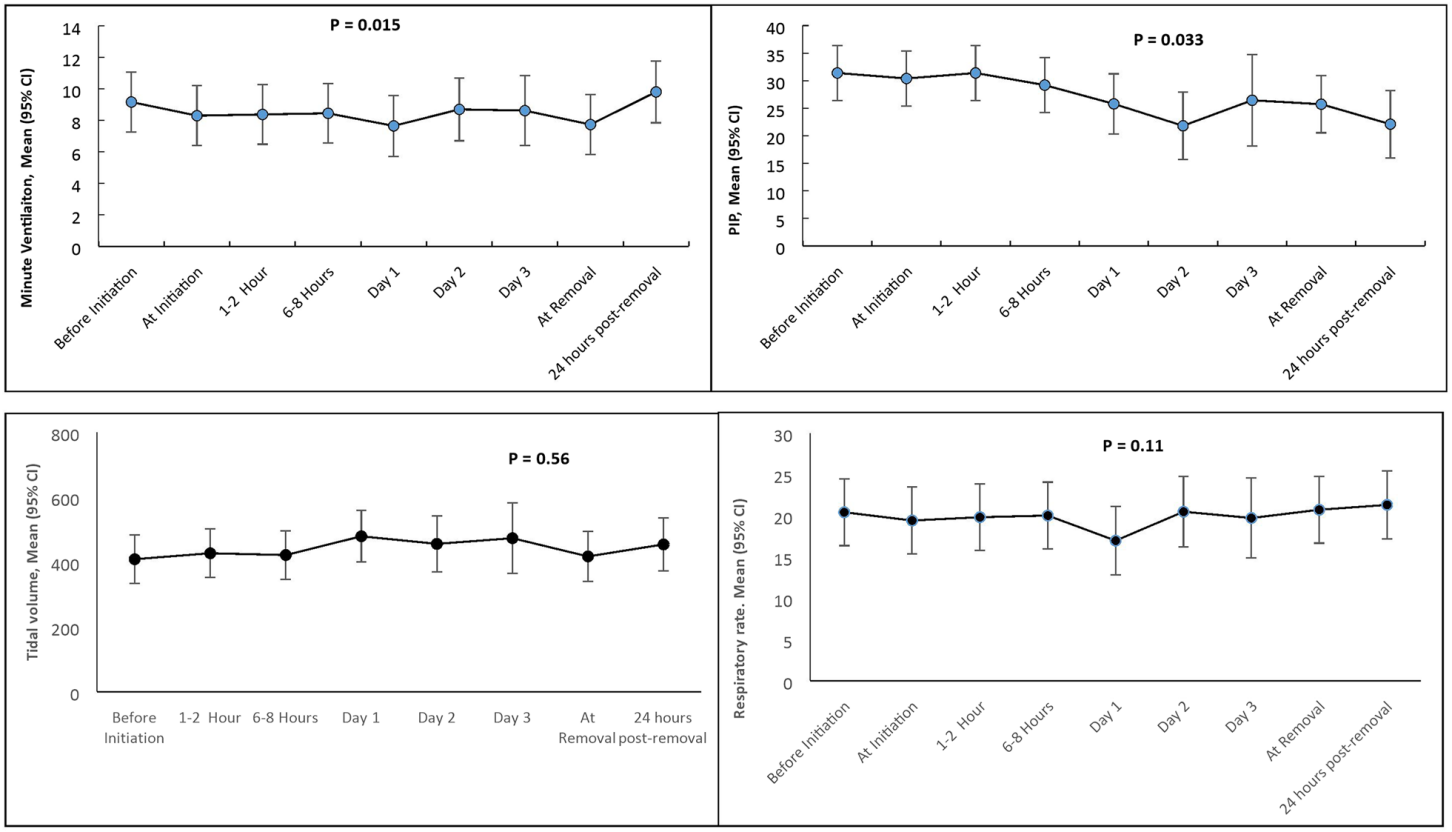
Changes in peak inspiratory pressure, minute ventilation, tidal volume and respiratory rate before initiation and at successive time points. Error bars represent 95% confidence intervals

A total of ten patients received ECCO_2_R during the study period. The demographic, diagnosis, and outcome data are presented in Table 1. A summary of the patients, including duration of ECCO_2_R, complications, survival and the cause of death is presented in Table 2. Figure 2 shows changes in pH and PaCO_2_ before initiation and at successive time points. Figure 3 shows changes in minute ventilation and peak inspiratory pressure before initiation and at successive time points. Table 3 shows mean changes in minute ventilation, peak inspiratory pressure, PaCO_2_, PaO_2_, pH, respiratory rate and tidal volume before initiation and at successive time points. A significant reduction in PaCO_2_ and improvement in pH were noted within 30 min of initiation of ECCO_2_R. These clinically important changes persisted throughout the therapy. The minute ventilation showed a reduction by day 1 of ECCO_2_R initiation. A reduction in peak inspiratory pressures was noted on day 2 after the initiation of ECCO_2_R.

**Table 1:** Summary of clinical characteristics and outcomes of patients receiving ECCO_2_R

Variable (*n* = 10 patients)	
Age in years (median; IQR)	55.5 (41–68)
Male, *n* (%)	8 (80%)
ARDS, *n* (%)	6 (60%)
COPD, *n* (%)	2 (20%)
Asthma, *n* (%)	2 (20%)
Serum Bilirubin (umol/L) (median; IQR)	11 (6–16)
Serum Albumin (g/L) (median; IQR)	30 (26–35)
Urea (mmol/L) (median; IQR)	15 (11–21)
Creatinine (umol/L) (median; IQR)	75 (64–118)
Hb (median; IQR)	11 (10–12)
Platelets (median; IQR)	234 (159–331)
WCC (median; IQR)	15 (11–21)
APACHE III score (median; IQR)	47(41–56)
Vascular access—femoral vein, *n* (%)	9 (90%)
Vascular access—femoral vein, *n* (%)	1 (10%)
Days on IMV prior to ECCO_2_R (median; IQR)	0.5 (0–1)
Days of IMV post ECCO_2_R (median; IQR)	4 (1–12)
Total duration of Mechanical ventilation (days) (median; IQR)	13.5 (6–26)
Total Duration of ECCO_2_R (hours) (median; IQR)	71 (57–219)
Total Duration of ICU stay (days) (median; IQR)	14.5 (8–30)
Number of ECCO_2_R kits used per patient (median; IQR)	1.5 (1–2)
Prone Position ventilation, *n* (%)	1 (10%)
Inhaled Nitric oxide, *n* (%)	1 (10%)
Survival to ECCO_2_R weaning, *n* (%)	9 (90)
Survival to ICU discharge, *n* (%)	8 (80)
Survival to hospital discharge, *n* (%)	7 (70)

**Table 2:** Summary of patients managed with PrismaLung+

Patient	Age	Sex	Diagnosis	Duration of ECCO_2_R (h)	Complications during ECCO_2_R	Survival to removal of ECCO_2_R	Survival to ICU discharge	Survival to hospital discharge	Cause of death
1	68	Male	ARDS	261	Circuit thrombosis < 72 h	Survived	Died	Died	Sepsis secondary to VAP
2	41	Male	ARDS	71	Circuit thrombosis < 72 h	Survived	Survived	Survived	–
3	37	Female	ASTHMA	69	Circuit thrombosis < 72 h	Survived	Survived	Survived	–
4	72	Male	ARDS	57	–	Survived	Survived	Died	Aspiration pneumonitis
5	43	Male	ARDS	31.5	Circuit thrombosis < 72 h	Survived	Survived	Survived	–
6	33	Male	ARDS	29	–	Survived	Survived	Survived	–
7	54	Female	COPD	219	–	Survived	Survived	Survived	–
8	57	Male	ASTHMA	71	–	Survived	Survived	Survived	–
9	78	Male	ARDS	275	–	Died	Died	Died	COVID-19 pneumonia
10	58	Male	COPD	89	–	Survived	Survived	Survived	–

**Table 3:** Mean changes in minute ventilation, peak inspiratory pressure, PaCO_2_, PaO_2_, pH, respiratory rate and tidal volume before initiation and at successive time points

Variable	Estimated change from before initiation	Standard error	*P *value
Minute ventilation			
At Initiation	0.870	0.503	0.090
1–2 h	0.790	0.503	0.123
6–8 h	0.720	0.503	0.159
Day 1	1.508	0.540	0.008
Day 2	0.464	0.595	0.439
Day 3	0.486	0.778	0.535
At Removal	1.447	0.519	0.008
24 h post removal	− 0.629	0.564	0.271
Peak inspiratory pressure (PIP)			
At Initiation	1.000	2.947	0.736
1–2 h	0.000	2.947	1.000
6–8 h	2.200	2.947	0.459
Day 1	5.599	3.160	0.083
Day 2	9.879	3.483	0.007
Day 3	6.007	4.541	0.193
At Removal	5.593	3.042	0.073
24 h post removal	9.288	3.479	0.011
PaCO_2_			
At Initiation	23.430	6.110	0.0004
1–2 h	29.580	6.110	< 0.0001
6–8 h	30.580	6.110	< 0.0001
Day 1	38.759	6.243	< 0.0001
Day 2	36.636	6.455	< 0.0001
Day 3	37.263	7.233	< 0.0001
At Removal	37.580	6.110	< 0.0001
24 h post removal	38.263	6.239	< 0.0001
PaO_2_			
At Initiation	3.033	10.188	0.767
1–2 h	14.756	10.188	0.155
6–8 h	17.956	10.188	0.085
Day 1	20.634	10.568	0.057
Day 2	1.628	10.838	0.881
Day 3	13.479	12.551	0.289
At Removal	18.211	10.188	0.081
24 h post removal	9.008	10.557	0.398
pH			
At Initiation	− 0.107	0.037	0.006
1–2 h	− 0.148	0.037	0.0002
6–8 h	− 0.166	0.037	< 0.0001
Day 1	− 0.237	0.039	< 0.0001
Day 2	− 0.211	0.041	< 0.0001
Day 3	− 0.204	0.048	0.0001
At Removal	− 0.244	0.037	< 0.0001
24 h post removal	− 0.271	0.039	< 0.0001
Respiratory rate			
At Initiation	0.999	1.056	0.348
1–2 h	0.599	1.056	0.573
6–8 h	0.399	1.056	0.707
Day 1	3.314	1.152	0.006
Day 2	− 0.172	1.293	0.894
Day 3	0.578	1.746	0.742
At Removal	− 0.30	1.056	0.778
24 h post removal	− 0.873	1.150	0.452
Tidal volume			
1–2 h	− 18.40	32.237	0.572
6–8 h	− 12.90	32.237	0.691
Day 1	− 69.591	35.078	0.055
Day 2	− 42.275	39.304	0.289
Day 3	− 62.782	52.829	0.242
At Removal	− 8.767	33.507	0.795
24 h post removal	− 47.836	36.90	0.203

There were no bleeding complications noted with the use of ECCO_2_R, but two patients required blood transfusions (one patient received four units and the other two units) largely due to loss of blood due to circuit changes and haemodilution. There were no other patient-related complications. A total of 18 circuits were used in ten patients (median 2 per patient; IQR 1–2). Circuit thrombosis was noted in five circuits (28%) that required replacement of the circuit prior to reaching the expected circuit life. It was due to a lack of anticoagulation at the time of initiation of ECCO_2_R, in one of these patients. No other device-related complications were noted. The survival to weaning of ECCO_2_R, ICU, hospital discharge, and cause of death are presented in Tables 1 and 2. Three patients required tracheostomy during their ICU course. All patients who survived were discharged home.

## Discussion

### Key findings

In this 10-patient case series to assess the efficacy of PrismaLung+ in correcting hypercapnic acidosis, we found that ECCO_2_R significantly improved hypercapnic acidosis within 30 min and maintained normal pH and normocapnia throughout the therapy while reducing minute ventilation and inspiratory pressure. There were no patient-related complications associated with the use of this device. Circuit thrombosis within 72 h of initiating ECCO_2_R was noted in four patients and was the only device-related complication that was noted in this study. The factors that may have contributed included poor vascular access that was noted in one patient due to the catheter inserted in the jugular vein and inadequate anticoagulation in another patient. Experience with the use of the device may help reduce the incidence of such complications.

### Relationship with previous studies

Several ECCO_2_R devices are currently in use with variable performances. PrismaLung+ is a novel device with the advantage of providing both ECCO_2_R and renal replacement therapy (RRT) with a single access catheter. The similarity of the device with RRT makes use of this device easier in intensive care units that currently use RRT. The results of our study in terms of CO_2_ removal are comparable to other studies with higher blood flows (350–550 mL/min) [16, 21]. This is due to the fact that the larger surface area of the membrane offsets the relative lower blood flow rates of 200–250 mL/min that were able to achieve with PrimaLung+  [25].

Our indication for the use of PrismaLung+ was different from some of the recent studies, where ECCO_2_R devices were used to target ultra-protective ventilation [9, 21]. Targeting ultra-protective ventilation did not improve 90-day mortality and was associated with lower ventilation-free days in patients with a PaO2/FiO2 of less than 150 mmHg [21]. Given these results, our aim was to first investigate if PrismaLung+ had the efficacy of removing CO_2_ with comparably lower flow rates (200 to 250 mL/min) when the low tidal volume (≤ 6 mL/kg of ideal body weight) caused hypercapnic acidosis. Our results suggest that PrismaLung+ was effective in the removal of CO_2_ and thereby correcting the hypercapnic acidosis associated with low tidal volume ventilation. The safety and efficacy of PrismaLung+ with ultralow tidal volume ventilation remain to be evaluated.

From the published data, ECCO_2_R devices while being effective in removing CO_2_, they have not been shown to improve survival, especially in patients with severe ARDS [9, 21]. Such patients with severe ARDS, are likely to benefit from VV ECMO [26]. Patients with mild to moderate ARDS associated with hypercapnic acidosis may benefit from low-flow ECCO_2_R devices [16].

The published literature on the use of this device is limited [20, 27, 28], with only one study reporting on the exclusive use of PrismaLung+ in mechanically ventilated patients with hypercapnic acidosis [27]. Similar to the results of our study, the study by Consales and colleagues reported a rapid correction of hypercapnic acidosis with no treatment-related complications [27]. The study by Giraud and colleagues [20] reported that PrismaLung+ was not able to remove sufficient CO_2_, to correct hypercapnic acidosis in three patients with severe COPD. In our case series, two patients had COPD, and PrismaLung+ satisfactorily improved hypercapnic acidosis.

In previous studies, low-flow ECCO_2_R devices were shown to have a higher proportion of haemolysis, bleeding, and membrane clotting as compared to high-flow ECCO_2_R devices [17, 19]. In our study, we did not find any similar clinical complications with PrismaLung+ such as bleeding or haemolysis other than the clotting of the circuit within 72 h after initiation of the ECCO_2_R in five circuits.

### Study implications and future directions

This study provides preliminary data on the safety and efficacy of ECCO_2_R with PrismaLung+ in mechanically ventilated patients. Further data on the efficacy of this device is required to determine whether it will reduce tidal volumes and driving pressure and thus improve survival in patients with ARDS.

### Strengths and limitations

*Strengths:* This study provides further evidence of the use of PrismaLung+ as an intervention to correct hypercapnic acidosis in patients receiving low tidal volume ventilation. The study results provide insights into the clinical efficacy and safety profile of the device that may help clinicians who may be considering the introduction of ECCO_2_R to their clinical practice. It reports data on physiological and patient-centred outcomes, especially the safety of this device.

### Limitations

Our study included patients, in whom lower tidal volumes were used. The efficacy of this device to provide satisfactory CO_2_ clearance in patients receiving ultralow tidal volumes was not evaluated in our study. We used an increase in serum bilirubin or anaemia that is not due to other obvious causes, as a marker of haemolysis. They may not be as sensitive as other investigations, such as haptoglobin or free haemoglobin for haemolysis. Given the single-centre experience of our study, the results may not be generalisable.

## Conclusions

ECCO_2_R with the use of PrismaLung+ appears safe, and effective in correcting hypercapnic acidosis. This data provides insight into PrismaLung+ performance and potential complications that could be useful for centres aspiring to introduce ECCO_2_R into their clinical practice. Further studies are required to evaluate its use in reducing driving pressure and associated lung injury, which may contribute to an improvement in clinical outcomes, including a reduction in the duration of mechanical ventilation and the associated morbidity and mortality.

## Data Availability

All data generated or analysed during this study are included in this published article.

## Abbreviations

ACT: Activated clotting time
APTT: Activated Partial Thromboplastin Time
ARDS: Acute respiratory distress syndrome
COPD: Chronic obstructive pulmonary disease
ECCO_2_R: Extracorporeal carbon dioxide removal
ECMO: Extracorporeal membrane oxygenation
ICU: Intensive care unit
IMV: Invasive mechanical ventilation
IQR: Interquartile range
PaCO_2_: Partial pressure of carbon dioxide in arterial blood
PaO_2_: Partial pressure of oxygen in arterial blood
RRT: Renal replacement therapy
VILI: Ventilator induced lung injury
